# Muscle Logic: New Knowledge Resource for Anatomy Enables Comprehensive Searches of the Literature on the Feeding Muscles of Mammals

**DOI:** 10.1371/journal.pone.0149102

**Published:** 2016-02-12

**Authors:** Robert E. Druzinsky, James P. Balhoff, Alfred W. Crompton, James Done, Rebecca Z. German, Melissa A. Haendel, Anthony Herrel, Susan W. Herring, Hilmar Lapp, Paula M. Mabee, Hans-Michael Muller, Christopher J. Mungall, Paul W. Sternberg, Kimberly Van Auken, Christopher J. Vinyard, Susan H. Williams, Christine E. Wall

**Affiliations:** 1 Department of Oral Biology, University of Illinois at Chicago, Chicago, Illinois, United States of America; 2 RTI International, Research Triangle Park, North Carolina, United States of America; 3 Organismic and Evolutionary Biology, Harvard University, Cambridge, Massachusetts, United States of America; 4 Division of Biology and Biological Engineering, M/C 156–29, California Institute of Technology, Pasadena, California, United States of America; 5 Department of Anatomy and Neurobiology, Northeast Ohio Medical University, Rootstown, Ohio, United States of America; 6 Oregon Health and Science University, Portland, Oregon, United States of America; 7 Département d’Ecologie et de Gestion de la Biodiversité, Museum National d’Histoire Naturelle, Paris, France; 8 University of Washington, Department of Orthodontics, Seattle, Washington, United States of America; 9 National Evolutionary Synthesis Center, Durham, North Carolina, United States of America; 10 Center for Genomic and Computational Biology, Duke University, Durham, North Carolina, United States of America; 11 Department of Biology, University of South Dakota, Vermillion, South Dakota, United States of America; 12 Genomics Division, Lawrence Berkeley National Laboratory, Berkeley, California, United States of America; 13 Howard Hughes Medical Institute, M/C 156–29, California Institute of Technology, Pasadena, California, United States of America; 14 Department of Biomedical Sciences, Ohio University Heritage College of Osteopathic Medicine, Athens, Ohio, United States of America; 15 Department of Evolutionary Anthropology, Duke University, Durham, North Carolina, United States of America; Brown University, UNITED STATES

## Abstract

**Background:**

In recent years large bibliographic databases have made much of the published literature of biology available for searches. However, the capabilities of the search engines integrated into these databases for text-based bibliographic searches are limited. To enable searches that deliver the results expected by comparative anatomists, an underlying logical structure known as an ontology is required.

**Development and Testing of the Ontology:**

Here we present the Mammalian Feeding Muscle Ontology (MFMO), a multi-species ontology focused on anatomical structures that participate in feeding and other oral/pharyngeal behaviors. A unique feature of the MFMO is that a simple, computable, definition of each muscle, which includes its attachments and innervation, is true across mammals. This construction mirrors the logical foundation of comparative anatomy and permits searches using language familiar to biologists. Further, it provides a template for muscles that will be useful in extending any anatomy ontology. The MFMO is developed to support the Feeding Experiments End-User Database Project (FEED, https://feedexp.org/), a publicly-available, online repository for physiological data collected from *in vivo* studies of feeding (e.g., mastication, biting, swallowing) in mammals. Currently the MFMO is integrated into FEED and also into two literature-specific implementations of Textpresso, a text-mining system that facilitates powerful searches of a corpus of scientific publications. We evaluate the MFMO by asking questions that test the ability of the ontology to return appropriate answers (competency questions). We compare the results of queries of the MFMO to results from similar searches in PubMed and Google Scholar.

**Results and Significance:**

Our tests demonstrate that the MFMO is competent to answer queries formed in the common language of comparative anatomy, but PubMed and Google Scholar are not. Overall, our results show that by incorporating anatomical ontologies into searches, an expanded and anatomically comprehensive set of results can be obtained. The broader scientific and publishing communities should consider taking up the challenge of semantically enabled search capabilities.

## Introduction

In recent years the construction of large bibliographic databases has made much of the published literature of biology available for searches. But, as any biologist who has performed online literature searches knows, the capabilities of the search engines indexing these databases are limited. Consider the following scenario: An investigator is interested in masticatory (chewing) muscles of mammals. She begins her work with a review of the literature. This turns out to be much more challenging than one might think. For example, the popular scientific literature search engines PubMed and Google Scholar return tens of thousands of hits for ‘masticatory muscle.’ However, only a small subset of these is relevant to her work, and many relevant articles are missed, because the results are limited to articles that contain the phrase “masticatory muscle” or the words “masticatory” and “muscle” in the title, abstract or in some cases, the full text. These and other primarily keyword index-driven search engines do not “know” what semantically a masticatory muscle is: a muscle that is active during mastication or, more broadly, feeding. To find papers that pertain to any of the masticatory muscles in mammals, the full list of those muscles would need to be entered in the query. Although such brute force methods are possible (e.g., [1]), they are tedious, prone to error by omission, and hardly available to anyone except those few with the requisite expert knowledge. In contrast, semantic searches, i.e., ones that take advantage of computationally available expert knowledge, are currently not supported by most comprehensive literature databases. For comparative anatomy, and for many other domains of knowledge, this is, in part, because the specialized body of anatomical knowledge is currently not represented in a computable form that can be used by such tools.

An ontology is a vocabulary of classes (also called ‘terms’) in which the classes, and the relationships between them, are well-defined based on *a priori* knowledge. This organization thereby reflects the knowledge of a domain in such a way as to be computable [2]. In an ontology, a term that has a computable definition is one that has one or more logical axioms that define the necessary and sufficient conditions that members of the corresponding class have to meet. A machine reasoner uses those axioms to infer relationships between classes. For example, ‘epiphysis of digit’ can be automatically classified (subsumed) as a subtype of ‘epiphysis of hand’ based on an axiom that ‘digit’ is a part of the ‘hand’, and similarly ‘epiphysis of distal phalanx’ can automatically be classified as a subtype of ‘epiphysis of digit’ [3].

In the past decade, there has been tremendous progress in the development of ontologies in biology [4, 5, 6]. The most well-known and frequently used and cited ontology in biology is the Gene Ontology (GO; [7]). The GO is used for gene product annotation that describes biological processes, molecular functions, and subcellular localization. There has also been comprehensive and pioneering work to develop species-centric anatomy ontologies for data on model organisms, such as the mouse developmental anatomy ontology (EMAPA; [8]), the adult mouse anatomy ontology (MA; [9]), the fly anatomy ontology (FBbt; [10]), and the zebrafish anatomy ontology (ZFA; [11]). Multi-species anatomy ontologies initially emerged to meet the needs of the evolutionary biology community. These included one for fishes [12], for amphibians [13], and one for vertebrates [14]. These have been recently merged into a taxonomically broader community anatomy ontology known as Uberon; [3, 6]. Uberon encompasses metazoan anatomy, with an emphasis on vertebrates, and is fully integrated with species-specific anatomy ontologies.

Here we present the Mammalian Feeding Muscle Ontology (MFMO), a multi-species anatomy ontology focused on muscles and associated anatomical structures that participate in feeding and other oral/pharyngeal behaviors. The MFMO was developed to support the Feeding Experiments End-User Database (FEED) Project ([15]; https://feedexp.org/). FEED is a publicly-available, online repository for physiological data collected from *in vivo* studies of feeding behaviors (i.e., oral/pharyngeal behaviors including mastication, biting, swallowing) of mammals. The FEED database facilitates quantitative and comparative studies of feeding and other behaviors in a manner that heretofore would have been difficult, if not impossible, as demonstrated by the promising preliminary analyses of Vinyard et al. [16] and Williams et al. [17] across Eutherian mammals.

To enable queries against FEED to return the most comprehensive, precisely focused, and relevant data possible to users, building FEED necessitated an ontological representation of the anatomical structures that participate in feeding behaviors, including relationships among muscles, their development, attachments, form and function. The resulting MFMO, which is incorporated into the search engine of the online FEED database, generalizes some of the concepts in Uberon [3, 6], and adds new terms and relations for muscle structures. In addition, the MFMO makes direct use of non-muscle classes from Uberon, such as classes for bones and nerves, that are required to define muscles. The eventual goal is to have MFMO be available as an extract or module of Uberon, but additional alignment needs to be completed.

In the pages that follow we describe the MFMO in detail, and we demonstrate its utility using a number of competency questions chosen to cover common comparative anatomy use-cases. We show that by using a minimum set of logical relationships in a search, a user can search efficiently and discover a comprehensive set of the relevant data. While obtained specific to the MFMO, one will likely find that these results generalize to any anatomy ontology and to any taxonomic group.

## Materials and Methods

### Initialization, Annotations, and Logical Definitions

**Fig 1:**
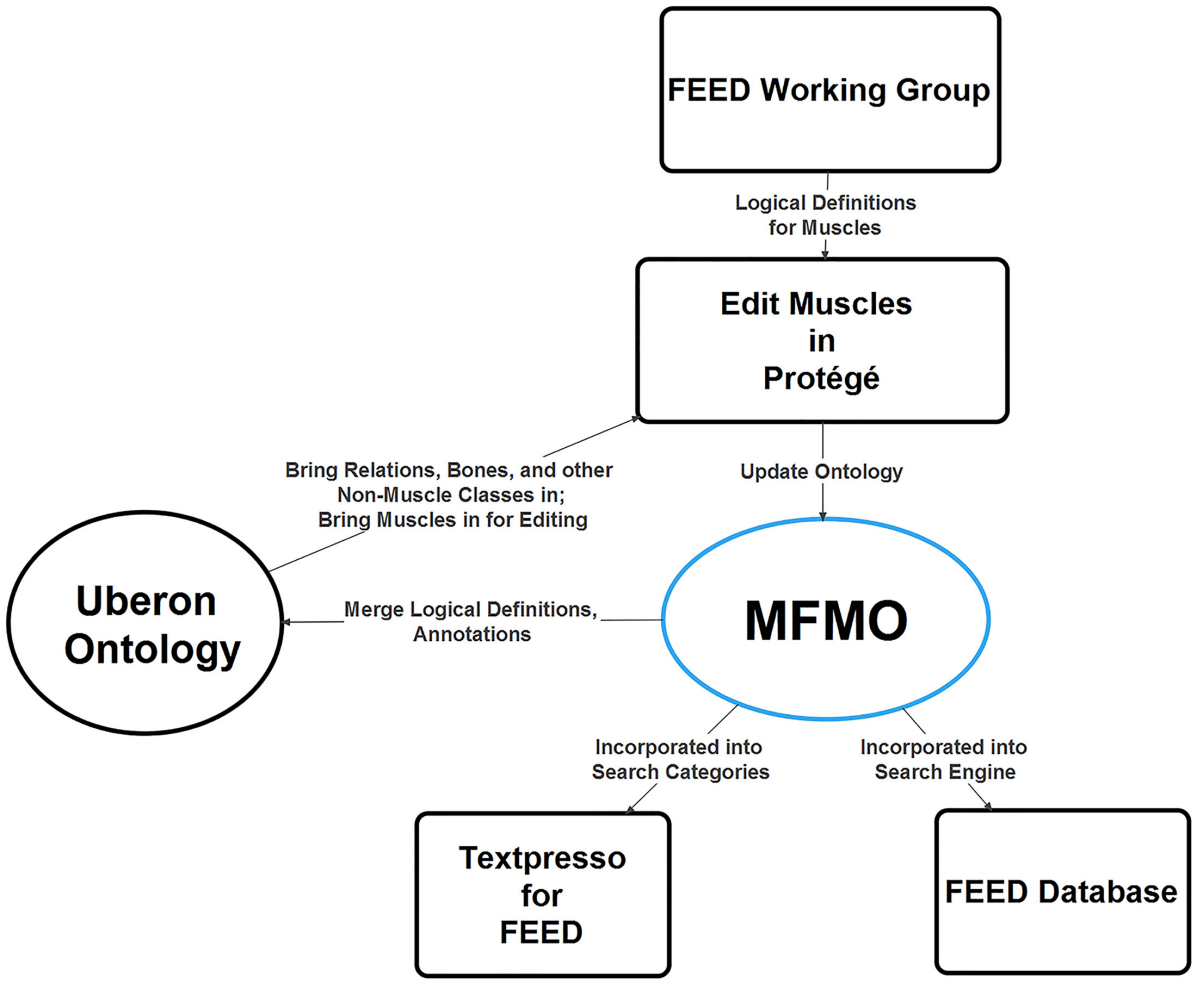
Work flow for the development of the MFMO and deployment for use cases. Creation of the MFMO began with the consensus definitions of the FEED Working Group, which were turned into logical definitions and then annotated in OWL using the Protégé software, with relations and classes imported from Uberon and other ontologies. The use-cases test the competency of the MFMO through queries in Protégé, FEED, and two Textpresso sites (see text).

The Mammalian Feeding Muscle Ontology (MFMO) includes the muscles that participate in oral/pharyngeal behaviors. A “generic” definition, true for all mammalian species, is given for each muscle. Each textual definition of a muscle is a consensus based on the literature of comparative anatomy and the collective knowledge of the authors, the FEED Working Group (Fig 1). (definition_source:FEED Working Group in the ontology). Some non-muscular structures, such as skeletal elements or nerves, have been imported from Uberon and several other ontologies. An anatomy ontology is an ontology of structures. At first, anatomy ontologies were dominated by is_a and part_of relations [18]. For example, a femur is_a bone and is part_of some lower limb. These relations are very useful. With them, one can search for all of the components in the lower limb, for example. But without other, biologically relevant relations, for example attached_to and innervated_by, such definitions are far too simplistic to be useful for contemporary biologists.

Higher-level anatomical classes have been imported from Common Anatomy Reference Ontology (CARO; [19]) and the Biological Spatial Ontology (BSPO; [20]). Most relations have been imported from the subset of the Relations Ontology (RO) used in Uberon ([6], supp.1; the ‘innervated_by’ relation is adapted from [21]; https://github.com/oborel/obo-relations). Because the MFMO is being used as a structured, controlled vocabulary and ontology module to maximize search capabilities within the FEED database [15], it was necessary to carefully refine the annotations and logical definition of each muscle class.

The MFMO ontology is maintained in a GitHub repository, where it is available for examination and download in Web Ontology Language (OWL) format (http://purl.obolibrary.org/obo/mfmo.owl). Each class is in the MFMO namespace, and is uniquely identified by a URI of the form: http://purl.obolibrary.org/obo/MFMO_nnnnnnn. MFMO is maintained and edited in Protégé (v. 5.0; http://protege.stanford.edu). We import images using the Image depiction Protégé plugin (https://github.com/balhoff/image-depictions-view). The MFMO uses an ontology ‘Continuous Integration server approach' (http://bio-ontologies.knowledgeblog.org/405) as described in Haendel, et al [6]. To promote expert review, the annotations and logical definition of each class are posted as tracker items for review by the FEED and Uberon communities in the GitHub repository for Uberon (https://github.com/obophenotype/uberon/issues).

For each muscle a class is created and given a unique ID (MFMO_nnnnnnn). The class is given a label, usually the most commonly used name for the muscle in the USA. A textual definition for each muscle is given, based on the consensus of the FEED Working Group with reference to the published literature. The source of the definition is provided (the FEED Working Group is cited as definition_source:FEED Working Group) as well as citations of significant literature. Whenever possible, an external reference (xref) for the class is provided, most often to an existing class in Uberon. Synonyms are added when they are not found in the Uberon class annotations. Other annotations added include comments regarding problematic definitions, notes about the existing definition in Uberon, and other issues that will help the ontology editor and inform users when the class is merged into Uberon. Nerves, bones, and other non-muscle entities are imported from Uberon. In the few cases in which these needed entities were not in Uberon, the class was added to FEED, to be added later to Uberon.

A logical definition is created for each muscle. The logical definition for each muscle takes the basic form:

innervated_by some nerve
attaches_to some entity A
attaches_to some entity B

For example, the styloglossus muscle (MFMO_0000066) is defined by the following axioms:

innervated_by some ‘hypoglossal nerve’ (MFMO_0000301)
attaches_to some ‘stylohyal bone’ (MFMO_0000053)
attaches_to some ‘tongue’ (MFMO_0000107)

This means that across mammals, any muscle that is innervated by the hypoglossal nerve, and that attaches to the stylohyal bone and the tongue is by definition a styloglossus muscle. The converse is true as well.

### Structure of the MFMO

To populate the MFMO we first considered taking a slice of the multi-species anatomy ontology, Uberon [3, 6], containing all of the classes for muscles and associated structures required for FEED, but that approach proved to be very inefficient. The initial version of Uberon was created through a system in which generalized classes were extracted from existing species-centric anatomy ontologies. Many of the textual definitions for muscles were extracted from Wikipedia via the DBpedia database [3, 22]. As a result, we found that most of the textual definitions for anatomical structures in Uberon were human-centric and the computable definitions too simple to make each one unique, necessary, and sufficient across mammals. Thus, this effort required creation of a new class for each muscle in the MFMO. A total of 58 classes for mammalian muscles have been created in the MFMO to date, all referenced to synonymous classes in Uberon (and other anatomy ontologies) with annotations.

**Fig 2:**
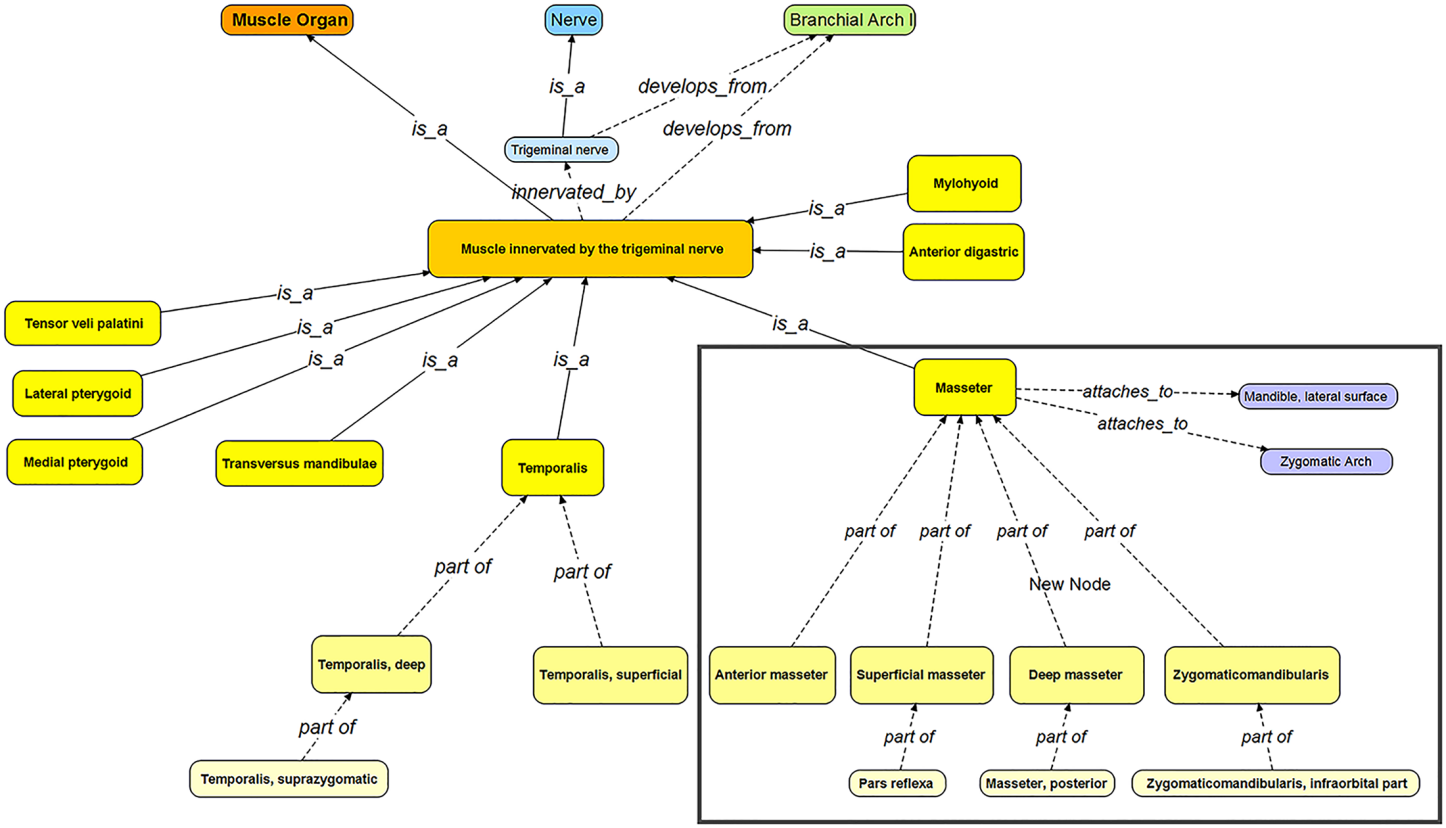
The structure of the trigeminal muscles in the MFMO. Ontological structure of muscles that develop from the first branchial arch/pharyngeal arch 1 and are innervated by the trigeminal nerve (“trigeminal muscles”) in the MFMO. The box indicates the masseter muscle, its parts, and its attachments. For simplicity, only the bony attachments of the masseter are shown.

In the MFMO, muscles are grouped into subclasses based on their innervation, which also infers their developmental origins. As an example, the muscles that are derived from the first branchial arch and their parts are presented in Fig 2. All of the muscles innervated by the trigeminal nerve are derived from branchial arch 1.

### Methods for Use-Cases

The value of the MFMO was assessed by asking competency questions [23] in three use-cases. We used the Description Logic Query function within Protégé (DL Query; http://protegewiki.stanford.edu/wiki/DLQueryTab) and the ELK reasoner [24] to assess the logical structure of the MFMO itself. We searched for trials (single physiological recordings) within the FEED Database to demonstrate the powerful search capabilities made available by building an anatomy ontology such as the MFMO into a database. Finally, we created Textpresso for FEED—a Textpresso demonstration site for the FEED project (http://textpresso-dev.caltech.edu/FEED/).

As explained in [25], a “competency question (CQ) is a natural language sentence that expresses a pattern for a type of question that people expect an ontology to answer. The answerability of CQs hence becomes a functional requirement of the ontology.” To test the competency of the MFMO “use-cases” the questions were formed in the familiar language of comparative biology to judge if the answers are appropriate in three settings: 1) in the MFMO ontology itself; 2) in the FEED online database; and 3) in Textpresso sites into which the MFMO is incorporated. Results of the CQs in MFMO are compared to similar searches in two popular databases, PubMed (http://www.ncbi.nlm.nih.gov/pubmed) and Google Scholar (https://scholar.google.com/), accessed through the University of Illinois, Chicago. Searches were performed between February and January of 2016.

To enable the FEED database, which is not integrated with any kind of reasoning capability, to utilize the MFMO for searching its records, we developed a tool that takes an OWL ontology (MFMO in our case) as input and, using the ELK reasoner, generates its transitive closure, which includes not only the asserted, but also all implied relationships, including those implied by recursion. In addition to computing the subclass hierarchy closure, the tool precomputes closures along selected transitive properties, such as “part of.” The tool outputs the result, a pre-reasoned ontology, in OWL format, which is then imported by an ontology import component that is part of FEED. The tool is available freely in source code form from GitHub (https://github.com/NESCent/feed-ontology-closure).

Textpresso is a text-mining system that facilitates powerful searches of a corpus of scientific literature (see [26] for a detailed description of the Textpresso system and construction of Textpresso sites). Textpresso allows users to query the full text of papers using keywords or semantically related categories (a shallow ontology; “bags of words”) or both, thus allowing highly specific queries for information retrieval from the literature. To create the Textpresso for FEED implementation, we compiled a corpus of 1636 articles in the domain of vertebrate functional evolutionary biology, primarily focused on the mammalian head and neck. PDFs were collected from the digital libraries of several members of the FEED Working Group and focus on the evolution, functional morphology, and developmental biology of the head and neck in mammals. No strict criteria were used for inclusion. Many articles were excluded because the text could not be interpreted by a text reader. The Textpresso for FEED corpus will be expanded to over 8000 articles in the near future. Going forward, it may be possible to automate the process of identifying and adding new literature to the corpus as it is published.

We created an ontology for the site by combining the MFMO, the Oral/Pharygeal Behavior Ontology (OPBO, an ontology of oral/pharyngeal behaviors, an ongoing project of the FEED Working Group; https://github.com/RDruzinsky/feedontology), a Eutherian slice of the Vertebrate Taxonomy Ontology (VTO; [27]) (placental mammals, such as humans, dogs, cows, bats, and their extinct relatives from the fossil record), and several existing Textpresso categories, such as those for human disease [28]. Textpresso for FEED refers to higher-order classes in the ontologies as ‘categories’. Sub-categories specific to Textpresso for FEED are found under the category “Biological Concepts.” Under Biological Concepts, the most relevant sub-categories are “bone,” “muscle,” “behavior,” and “mammal” (S1 Fig).

To process the corpus for searching, PDF files of each of the 1636 articles, with full bibliographic references, were first converted to plain text. The resulting text was then indexed for the location of all terms as well as the inclusion of terms in each of the Textpresso ontologies or categories. The Textpresso corpus thus contains full text with words and phrases labeled using the eXtensible Markup Language (XML), according to the lexicon of the ontology. Such indexing allows for rapid searches for sentences containing one or more desired keywords or ontology terms.

In addition to creating the Textpresso for FEED site, we also added the categories from the MFMO muscle and the OPBO to an existing Textpresso site, Textpresso for Mouse. As of July 2015, Textpresso for Mouse (http://www.textpresso.org/mouse/) contains almost 114,000 indexed papers on the “model organism” mouse.

## Results

The type of data returned by a query is dependent upon the data in the database, so results of queries in the use-cases are not precisely the same. Queries of the MFMO in Protégé return only the muscles (classes) themselves. Queries of the Textpresso sites return published articles, as do queries of PubMed and Google Scholar. Queries of FEED return trials (single recordings) in the database. All of the websites cited herein were active at the time of submission. We invite our readers to explore the sites and create their own queries.

### The MFMO facilitates searches for a muscle and synonyms

The MFMO includes synonyms for muscles. The MFMO underlies the FEED Database, thus recordings from any of the muscles in FEED can be retrieved by searching for a given muscle or any of its synonyms. The current FEED database is small (1069 trials as of 1/1/2016, and most of the data are, at present, electromyographic (EMG) recordings from trigeminal muscles. Even so, the competency of the ontology is apparent. If we ask FEED to find all recordings from the ‘lateral pterygoid muscle,’ FEED returns 31 trials whether we ask for the ‘lateral pterygoid muscle’ or its synonym, the ‘external pterygoid muscle’ (S2 Fig).

In contrast, PubMed and Google Scholar yield very different results when asked for papers that contain the text ‘lateral pterygoid muscle’ or ‘external pterygoid muscle.’ Searches return 773 papers for lateral pterygoid muscle and only 110 papers for external pterygoid muscle in PubMed. In Google Scholar the same searches return approximately 26,000 papers for lateral pterygoid (S3 Fig) and 25,700 papers for external pterygoid (S4 Fig). The reason that these searches return different numbers of papers for the same muscle is that these search engines do not synonymize the terms “lateral pterygoid muscle” and “external pterygoid muscle.” FEED returns appropriate answers to the questions; search engines on the large databases do not.

### The MFMO facilitates searches for muscles attached to a given bone in Protégé

We can also ask Protégé for all of the muscles that are attached to a given bone. For example, if we ask for all of the muscles attached to the mandible, [Protégé DL query: ‘muscle organ’ and (‘attached to’ some ‘mandible’)], Protégé returns the muscles listed in Table 1. This list is correct and once again the MFMO gives an appropriate answer to the question.

**Table 1:** Classes returned by the MFMO from a DL Query in Protégé for muscles attached to the mandible.

Muscles attached to the Mandible
Anterior digastric muscle
Buccinator muscle
Depressor anguli oris muscle
Depressor labii inferioris muscle
^+^Eutherian genioglossus muscle
Geniohyoid muscle
Lateral pterygoid muscle
Masseter muscle
Anterior masseter muscle
Deep masseter muscle
Posterior masseter muscle
Superficial masseter muscle
Masseter muscle, pars reflexa
Zygomaticomandibularis muscle	
Zygomaticomandibularis muscle, infraorbital portion
Medial pterygoid muscle
Mentalis muscle
Mylohyoid muscle
^+^Orangutan posterior digastric muscle
Orbicularis oris muscle
Platysma muscle
Temporalis muscle
Deep temporalis muscle
Superficial temporalis muscle
Suprazygomatic portion of the temporalis muscle

Protégé DL query of the MFMO: ‘muscle organ’ and ‘attached_to’ some mandible (Protégé returns a list of muscles attached to the mandible. Hierarchical indentation indicates that those muscles are part_of the larger muscle.

*MFMO uses the label ‘Eutherian genioglossus muscle’ to distinguish this muscle from the non-homologous ‘genioglossus’ muscles of other tetrapods.

^+^ ‘Orangutan posterior digastric muscle’ is a subclass of ‘posterior digastric muscle’ in that it uniquely attaches to the mandible rather than to a common tendon with the anterior digastric muscle [29].

If we ask PubMed for papers that include references to ‘muscles attached to mandible’ a very limited number of papers, i.e., 137, are returned (S5 Fig). The results are publications that contain the phrases ‘muscle,’ ‘mandible’ and ‘attached to’ in the title or abstract, not publications that specifically reference muscles that are attached to the mandible. For example, the search found Haddock, DeLacure,and Saadeh (2008), entitled “Functional reconstruction of glossectomy defects: the vertical rectus abdominus myocutaneous neotongue” [30] because the keyword terms and phrases are in the abstract:

“The **muscle** inset was supported at the inferior mandibular border **attached to** the remaining lingual mucosa or gingiva.”

“This neotongue sits on the **mandible** under voluntary control….”

Although this paper describes a surgical procedure in which a neotongue (a surgically manufactured, artificial tongue) is attached to the mucosa of the mandible, it is not about any of the muscles that are attached to the mandible. In addition, among the 137 references returned by PubMed there are two that contain the term ‘genioglossus muscle,’ which is a muscle attached to the mandible, but a search for the ‘genioglossus muscle’ in PubMed returns 863 papers. If the previous search had been for papers that contain any reference to any of the muscles that attach to the mandible, it would have found all 863 papers that contain the term ‘genioglossus.’ Similarly, a search for ‘muscle attached to mandible’ in Google Scholar returns 57,900 references of which 4,360 also contain the term ‘genioglossus.’ However, a search for ‘genioglossus muscle’ by itself returns 12,900 references. In other words the answers by PubMed and Google Scholar are, once again, inappropriate.

### The MFMO facilitates searches for muscles as grouped by comparative anatomists

If we query the MFMO for all of the muscles that are derivatives of the first branchial arch, [Protégé DL query: ‘muscle organ’ and (‘develops from’ some ‘branchial arch 1’)], Protégé returns the muscles listed in Table 2. The muscles in Table 2 and Fig 2 are the same. Thus, the answer given by the MFMO to the question asked is appropriate. In FEED, if we ask for all of EMG recordings from any ‘muscle innervated by the trigeminal nerve’ FEED returns 1066 of the total 1069 trials, and the muscles for which trials are returned in the search are appropriate.

**Table 2:** Classes returned by the MFMO from a DL Query in Protégé for muscles that develop from the first branchial arch.

Trigeminal muscle
Anterior digastric muscle
Lateral pterygoid muscle
Masseter muscle
Anterior masseter muscle
Deep masseter muscle
Posterior masseter muscle
Superficial masseter muscle
Superficial masseter, pars reflexa
Zygomaticomandibularis muscle
Zygomaticomandibularis, infraorbital portion
Medial pterygoid muscle
Mylohyoid muscle
Temporalis muscle
Deep temporalis muscle
Superficial temporalis muscle
Suprazygomatic portion of the temporalis muscle
Transversus mandibulae muscle
Tensor tympani muscle
Tensor veli palatini muscle

These muscles are known collectively as the ‘muscles innervated by the trigeminal nerve’ or the ‘trigeminal muscles.’ Hierarchical indentation indicates that those muscles are part_of the larger muscle.

If we ask Textpresso for FEED to retrieve papers that mention any trigeminal muscle by searching for “muscle innervated by the trigeminal nerve” (Table 2; see S7 Fig), it retrieves 452 documents. If we narrow the search by adding the category ‘mastication’ Textpresso finds 91 documents. Among these papers is Herring et al. (2011) entitled “Mastication and the postorbital ligament: dynamic strain in soft tissues” [31]. We can ask the same question in PubMed and Google Scholar by searching for “muscle innervated by the trigeminal nerve” and “mastication.” A search of PubMed returns 11 articles but not Herring et al. [31], even though the title includes the word “mastication” and the article is indexed in PubMed, as evidenced by its PMID (20235321). A search of Google Scholar for “muscle” and “mastication” returns 34,900 papers and Herring et al. [31] is among them, but a search for “muscle innervated by trigeminal nerve” and “mastication”, finds approximately 8160 articles but not Herring et al. [31]. The reason that this article is found in the search of Textpresso for FEED but not found in the concise PubMed or Google Scholar searches is that the Textpresso search includes “temporalis muscle” among the search terms, because it is a trigeminal muscle. A search in PubMed for ‘trigeminal’ and ‘muscle’ returns 1945 references, but a search for ‘temporalis muscle’ returns 10,593 references. The ‘temporalis muscle’ papers should be a subset of the ‘trigeminal muscle’ papers. In fact, the two searches have only 270 references in common. PubMed and Google Scholar do not “know” that the temporalis muscle is one of the muscles innervated by the trigeminal nerve.

We can refine the previous search to query only for trigeminal muscles that are attached to the mandible, [Protégé DL query: ‘muscle organ’ and (‘innervated by’ some ‘trigeminal nerve’) and (‘attached_to’ some ‘mandible bone’)]. Protégé returns the muscles listed in Table 3, which is the correct response (S6 Fig). A similar search of PubMed for ‘trigeminal muscle’ or ‘muscle innervated by trigeminal nerve’ and ‘attached to mandible’ returns only five publications. However, if we search for papers that contain any of these muscles (for simplicity, the searches were limited to the masseter, lateral pterygoid, medial pterygoid, temporalis, and anterior digastric muscles), we find 15,435 publications in PubMed. And, it should be noted, such searches do not include the synonyms of the muscles. So, the response from PubMed is not appropriate because the results (five papers) are far from comprehensive.

**Table 3:** Classes returned from a DL Query for muscles innervated by the trigeminal nerve that are attached to the mandible.

Trigeminal Muscles attached to the Mandible
Anterior digastric muscle
Lateral pterygoid muscle
Masseter muscle
Anterior masseter muscle
Deep masseter muscle
Posterior masseter muscle
Superficial masseter muscle
Superficial masseter, pars reflexa
Zygomaticomandibularis muscle
Zygomaticomandibularis, infraorbital portion
Medial pterygoid muscle
Temporalis muscle
Deep temporalis muscle
Superficial temporalis muscle
Suprazygomatic portion of the temporalis muscle
Transversus mandibulae muscle

Protégé DL query of the MFMO: ‘muscle organ’ and ‘innervated by’ some ‘trigeminal nerve’ and ‘attached to’ some ‘mandible bone.’ Hierarchical indentation indicates that those muscles are part_of the larger muscle.

We can also demonstrate the competency of the MFMO by searching the Textpresso for Mouse site that has the MFMO installed in the underlying ontology. Textpresso for Mouse (http://www.textpresso.org/mouse) is a Textpresso site that is focused on the literature pertaining to the “model organism” mouse, *Mus musculus*. At present, the corpus contains the full text of almost 114,000 papers. A search limited to articles that contain text references to the class “muscle innervated by the trigeminal nerve” returns 368 documents. When the search is narrowed by searching for papers that contain references to both the trigeminal muscles and terms from the Gene Ontology (GO) that refer to “molecular function,” the search finds 62 documents. An example of a paper found by this search is Katayama, Yamane, and Fukui (2010) entitled “Changes in the expression of myosins during postnatal development of masseter muscle in the microphthalmic mouse” [32]. The text matched in this paper was: masseter muscle, insulin-like growth factors, receptors, IGF binding proteins, myosin, and receptor. Similar searches in PubMed, and in Google Scholar return two papers and approximately 26,200 papers respectively, but the Katayama, Yamane, and Fukui [32] paper is not among them. PubMed and Google Scholar do not “know” that the masseter muscle is one of the muscles innervated by the trigeminal nerve, nor do these search engines “know” any of the terms related to molecular functions. The PubMed and Google Scholar searches only found papers in which the terms “muscle,” “innervated,” “trigeminal,” “nerve,” “molecular,” and “function” are present.

## Discussion

The use-cases described above demonstrate how the knowledge encoded in the MFMO enables databases such as FEED and Textpresso to index their content such that it can be retrieved in response to, and consistent with, language and terminology familiar to comparative anatomists. One can ask, for example, which muscles that develop from the second branchial arch are attached to the hyoid apparatus, because the requisite knowledge about developmental origin and attachments is encoded in the MFMO by means of logical axioms. We can also infer the answer to the question “from which branchial arch is the posterior digastric muscle derived?” The MFMO infers that the posterior digastric muscle is derived from the second branchial arch, because it is innervated by the facial nerve. This type of inference is an essential part of the logic of comparative anatomy.

The MFMO, like all ontologies, is not static. Rather, it is designed to evolve as new terms and relations are determined. Its use within FEED and in other contexts will facilitate future modifications of MFMO. Similarly, the integration of the MFMO into FEED will facilitate our efforts to synthesize and integrate physiological data, including the development of novel analytical tools and novel studies of key missing taxa.

Taxonomic bias and missing data are persistent problems in inter-specific analyses of physiologic data. Currently, the available data in FEED is heavily biased toward primates. This is due to the comparatively large number of primate species studied using *in vivo* experimental methods in comparison to other mammalian orders. Missing data must always be accounted for in statistical analyses and subsequent interpretations of biological phenomena.

FEED is a “proof-of-concept” database that is meant to grow as the data on mammalian and reptilian feeding grows. Analyses are currently underway that will identify the most critical taxonomic gaps in FEED. It is our hope, and a goal of the FEED project, that our colleagues who study mammalian feeding will also begin to use FEED and upload some of their data to the site. The MFMO is of central importance as it facilitates the computational comparisons that allow meaningful analyses of phenotypic diversity. Use of the MFMO in conjunction with the orthogonal ontology on oral/pharyngeal feeding behaviors (the OPBO) that is incorporated into FEED permits the integration of anatomical, physiological, and behavioral data so that the links between structure, function, and behavior can be explored in a systematic way across users and across disciplines.

For centuries, anatomists have named and distinguished muscles from one another on the basis of their attachments and innervations (e.g., [33], pages 362–3). The anterior digastric and geniohyoid muscles both attach to the mandible and the hyoid. Although one can distinguish between the two muscles by their specific attachments to the bones, they can also be distinguished on the basis of their innervations. The anterior digastric muscle is innervated by a branch of the trigeminal nerve and the geniohyoid muscle is innervated by a branch of the first cervical nerve (C1). For most of the oral/pharyngeal muscles of mammals, the details of the topography of the attachments to the bones are not required to distinguish between them. Hence, a broad definition need only specify the bones to which the muscle is attached and the innervation.

The literature of comparative anatomy and textbooks of human anatomy contain detailed descriptions of muscles in thousands of species. These descriptions are usually specific to a single species or a few closely related species. For example, a popular contemporary textbook of human anatomy [34] describes the hyoglossus as follows:

“The hyoglossus muscles are thin quadrangular muscles lateral to the genioglossus muscles….

“Each hyoglossus muscle originates from the entire length of the greater horn and the adjacent part of the body of the hyoid bone. At its origin from the hyoid bone, the hyoglossus muscle is lateral to the attachment of the middle constrictor muscle of the pharynx. The muscle passes superiorly and anteriorly through the gap (oropharyngeal triangle) between the superior constrictor, middle constrictor, and mylohyoid to insert into the tongue lateral to the genioglossus and medial to the styloglossus.

“The hyoglossus muscle depresses the tongue and is innervated by the hypoglossal nerve [XII]” [34], pages 1098–99.

In other taxa the hyoglossus is described differently. For example, the following is from a comparative study of New World squirrels [35]:

“The hyoglossus is a flat muscle that has a broad origin on the lateral surface of the posterior cornu of the hyoid and the anterior surface of the basihyal. On the basihyal, it originates dorsal to the insertion of the mylohyoid and geniohyoid. Its fibers pass dorsally, anteriorly, and laterally, and enter the ventro-lateral surface of the tongue…” [35], pages 283–4.

Strikingly, neither description starts with an explicit definition of the generalizable, necessary, and sufficient properties because of which the muscle in question is being referred to as the hyoglossus to begin with. Instead, this definition is almost always implicit. Not only is the reader assumed to know what it is, but it is also assumed that there is broad consensus about it.

In contrast to published descriptions such as the two above, the hyoglossus in the MFMO (MFMO_0000064) has a succinct, logical, and machine-interpretable definition:

('attached to' some 'hyoid bone')
and ('attached to' some tongue)
and ('innervated by' some 'hypoglossal nerve')

This definition contains only the necessary and sufficient conditions to decide whether a given muscle is a hyoglossus muscle, and thus the conditions that must be true for any muscle declared as or referred to as hyoglossus muscle (within Eutheria). This form of definition is used for all definitions of muscles in the MFMO. Obviously, the highly detailed descriptions of muscles in the literature are valuable and, ultimately, these details can be included in the MFMO as sub-classes of the muscles. But the use-cases elaborated above show that such succinct, generic, logical definitions as in the MFMO can be powerful as well. Interestingly, in practice students of anatomy learn quickly that a muscle can often be identified without examination of the details of the muscle attachments. In contemporary textbooks, the basic attachments, innervations, and functions of muscles are often summarized in large tables (e.g., [34]), Table 8.21).

Much progress has been made toward the development of a methodology for encoding phenotypic variation in ontologies and, using these new methods, the creation of databases with descriptions of taxa and individual specimens (e.g., [36, 37, 38]). Knowledge of anatomy encoded in machine-interpretable ontologies with concise logical definitions is not only powerful for intelligent information retrieval, but also will play a foundational role in transforming the rich descriptions of phenotypic variation into fully computable logic expressions based on the so-called ‘Entity–Quality’ (EQ) formalism [39]. In this formalism, a phenotype is modeled as a phenotypic quality, typically drawn from the Phenotype And Trait Ontology (PATO; [39]) that inheres in (is borne or possessed by) an anatomical entity (in the case of anatomical phenotypes), which is drawn from a requisite anatomy ontology.

EQ statements [40] are similar to, but not precisely the same as, character states, which are familiar to evolutionary biologists. The character state “eye color: blue” for example, is equivalent to the EQ statement “Entity: eye; Quality: blue.” The anatomical entity ‘eye’ possesses quality ‘blue’, which is a type of color [41]; the relation (blue is_a color) is a statement in the logical structure of the ontology. Software to facilitate EQ coding of phenotypic descriptions (Phenex; [41, 42]) has been developed. Ontologies that include axiomatic definitions of their classes, such as the MFMO for the muscles it contains, allow rich reasoning and computational assessment of semantic distance between phenotypes. Methods are being developed that, albeit still in their infancy, show great promise for utilizing anatomy and other ontologies for automated extraction and logical transformation of phenotype descriptions from pertinent literature [37, 40, 43, 44, 45].

The MFMO greatly benefits from, and extends a growing and rich ecosystem of biological ontologies that covers biological knowledge for a multitude of knowledge domains and several levels of granularity [46]. To facilitate consistency across ontologies, MFMO takes advantage of the Relation Ontology (RO; [18], suppl. Table 2) for relationships (properties); the Biological Spatial Ontology (BSPO; [19]) for spatial relations; the Uberon ontology ([3, 6]) as the major multi-species ontology for metazoan anatomy, which itself depends on the Common Anatomy Reference Ontology (CARO; [16]) for ensuring cross-ontology consistency of high-level anatomical classes; and the Vertebrate Taxonomy Ontology (VTO; [27]) for taxonomic restrictions of axioms.

Other recent work has focused on tools for reasoning across taxa and integration of disparate multi-species anatomy ontologies [47, 48, 49, 50] that permit queries about genes, development, and diseases. The most comprehensive effort of this type is Uberon [3, 6]. Uberon integrates anatomy ontologies by creating generalized definitions for classes that are true for higher taxa, cross references (xrefs) to terms in other ontologies, and “bridging extensions” that take classes from other ontologies but revise and broaden the definitions to make them applicable for higher taxa. However, expansion of Uberon is slow because construction of “generic” logical definitions for anatomical structures that are true at higher taxonomic levels, similar to those for the muscles in the MFMO, is extremely difficult.

The MFMO is a critical piece of the knowledge representation for vertebrate anatomy, transforming unstandardized text-based descriptions of muscle anatomy into computable anatomy. A simple definition for each muscle, one that includes its innervation and attachments, serves as the template for computable definitions of muscles. We have now begun to create concise, logical definitions for post-cranial muscles in Uberon, and we are also expanding the MFMO beyond Eutheria.

## Conclusions

Retrieving published knowledge from the vast online stores of literature, as illustrated by the opening scenario of a young investigator searching for the masticatory muscles of mammals, is one of the most common exercises in scientific pursuits, whether, as here, in comparative anatomy or in other fields. In contrast to keyword indexed search engines such as PubMed and Google Scholar, our results show how ontology-driven semantic searches such as those enabled by the MFMO can reduce the number of inappropriate articles returned, and find many articles that would otherwise have been missed. Although at present the MFMO and other pertinent ontologies have not been built into any of the major literature search engines, recently developed tools such as FEED [15] and Aber-OWL [48] show how the logical reasoning enabled by rich ontologies can allow machines to understand the semantics behind a query expression. More widely enabling semantics-aware searching is a future challenge for the broader scientific and publisher community.

The FEED and Textpresso sites with the MFMO installed make semantic searches possible, but they also do more than that. Both FEED and Textpresso enable the utilization of “dark data.” “Dark data” are defined as “any data that is not easily found by potential users” ([51], page 281). FEED is a repository for data on the physiology of feeding that resides in laboratories around the world. FEED will enable comparative studies across disparate species from these data. Textpresso and other projects such as the Paleobiology Database (https://www.paleobiodb.org/), attempt to make portions of the “dark” published literature of science available online in searchable form.

As argued by Wächter et al. [52], the literature holds the answers to many questions that a researcher might ask, “but a classical literature search cannot answer the questions directly.” Literature search indexes, such as PubMed and Google Scholar, lack the ability to utilize domain knowledge for query expansion, reduction, and filtering. In other words, tedious enumeration of concepts relevant to queries is required (e.g., listing all of the masticatory muscles), as well as expert knowledge of the subject. Conversely, if the domain knowledge is encoded in a computationally interpretable form in an ontology, databases can utilize it to expand queries based on logical entailments of query expressions, and ontology-powered query answering is then available to experts and non-experts alike.

The MFMO encodes the broad consensus of comparative anatomy knowledge about the head and neck muscles involved in feeding across Eutheria. It sets a standard for defining muscles in a succinct form that machines can reason with, including across the published literature. The merits of the MFMO are shown by its ability to satisfy a variety of competency questions covering and built from the language of common comparative biology. It is ground-breaking in several aspects: it is the first anatomy ontology of muscles in which there are logical definitions that are true across a major clade (Eutheria), and it includes new logical relationships specified by the attachments, development, and innervations of muscles. It is our hope that the template for logical definitions of muscles in the MFMO will be utilized in other anatomy ontologies and that these ontologies will one day serve to enable semantic querying capabilities in major bibliographic search engines.

## Supporting Information

S1 Fig Screenshot of Textpresso for FEED category menus. The muscles of the MFMO are found under the cascading drop-down menus: lists/Biological Concepts/muscles. The hierarchy of the Vertebrate Taxonomy Ontology are under: lists/Biological Concepts/mammal.
(PNG)

S2 Fig Screenshot of search for recordings from the lateral pterygoid muscle in FEED. Only the first page is displayed.
(PNG)

S3 Fig Screenshot of a search for lateral pterygoid in Google Scholar. Only the first page is displayed.
(PNG)

S4 Fig Screenshot of a search for external pterygoid in Google Scholar. Only the first page is displayed.
(PNG)

S5 Fig Screenshot of a search in PubMed for muscles attached to the mandible. Only the first page of results are displayed.
(PNG)

S6 Fig Screenshot of a DL Query in Protégé for muscles innervated by the trigeminal nerve that are attached to the mandible. (PNG)

S7 Fig Screenshot of the results from a query in Textpresso for FEED. Only the first page of results are displayed.
(PNG)
