# Supplementary figures and images for: Muscle Logic: New Knowledge Resource for Anatomy Enables Comprehensive Searches of the Literature on the Feeding Muscles of Mammals
Source: PLoS One. 2016 Feb 12;11(2):e0149102. doi: 10.1371/journal.pone.0149102 (PMC4752357; doi:10.1371/journal.pone.0149102)

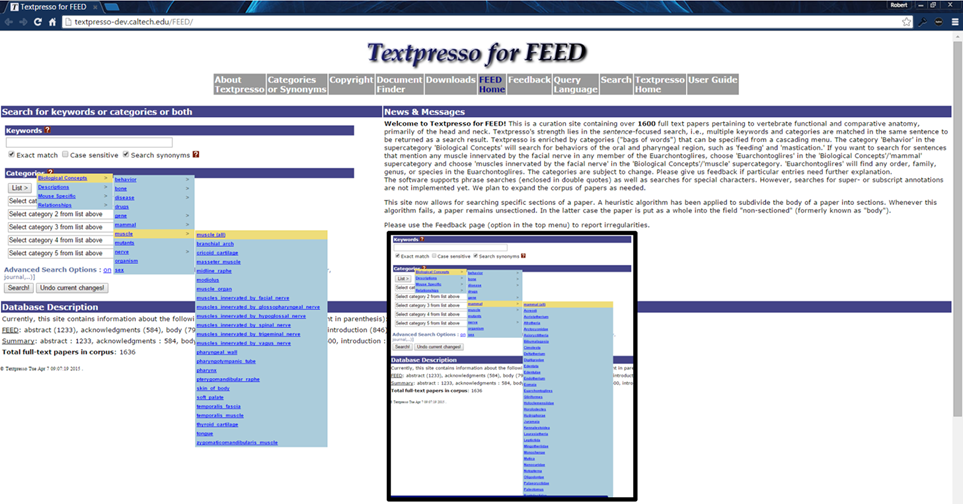

Supplement: S1 Fig — The muscles of the MFMO are found under the cascading drop-down menus: lists/Biological Concepts/muscles. The hierarchy of the Vertebrate Taxonomy Ontology are under: lists/Biological Concepts/mammal. (PNG) [file pone.0149102.s001.png]

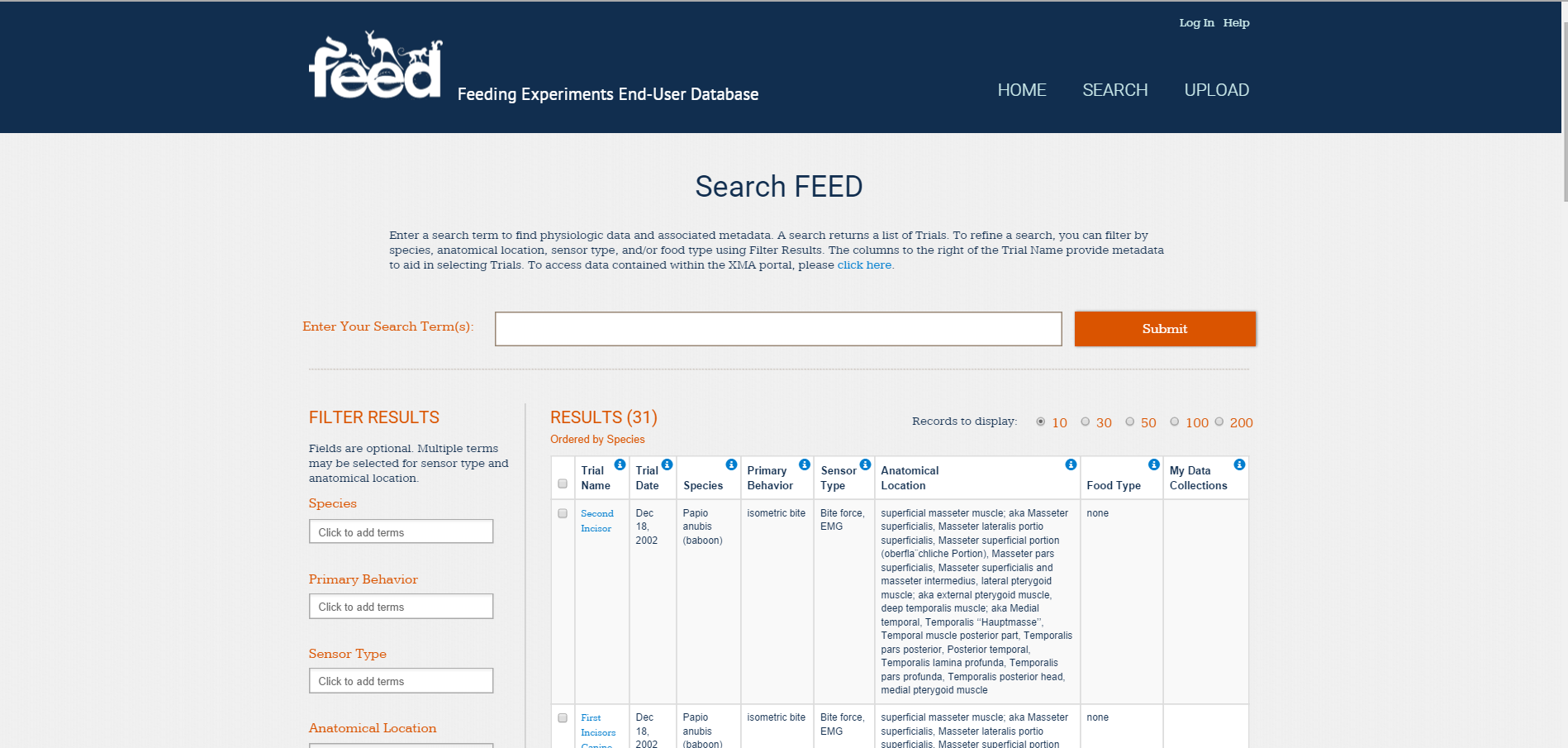

Supplement: S2 Fig — Only the first page is displayed. (PNG) [file pone.0149102.s002.png]

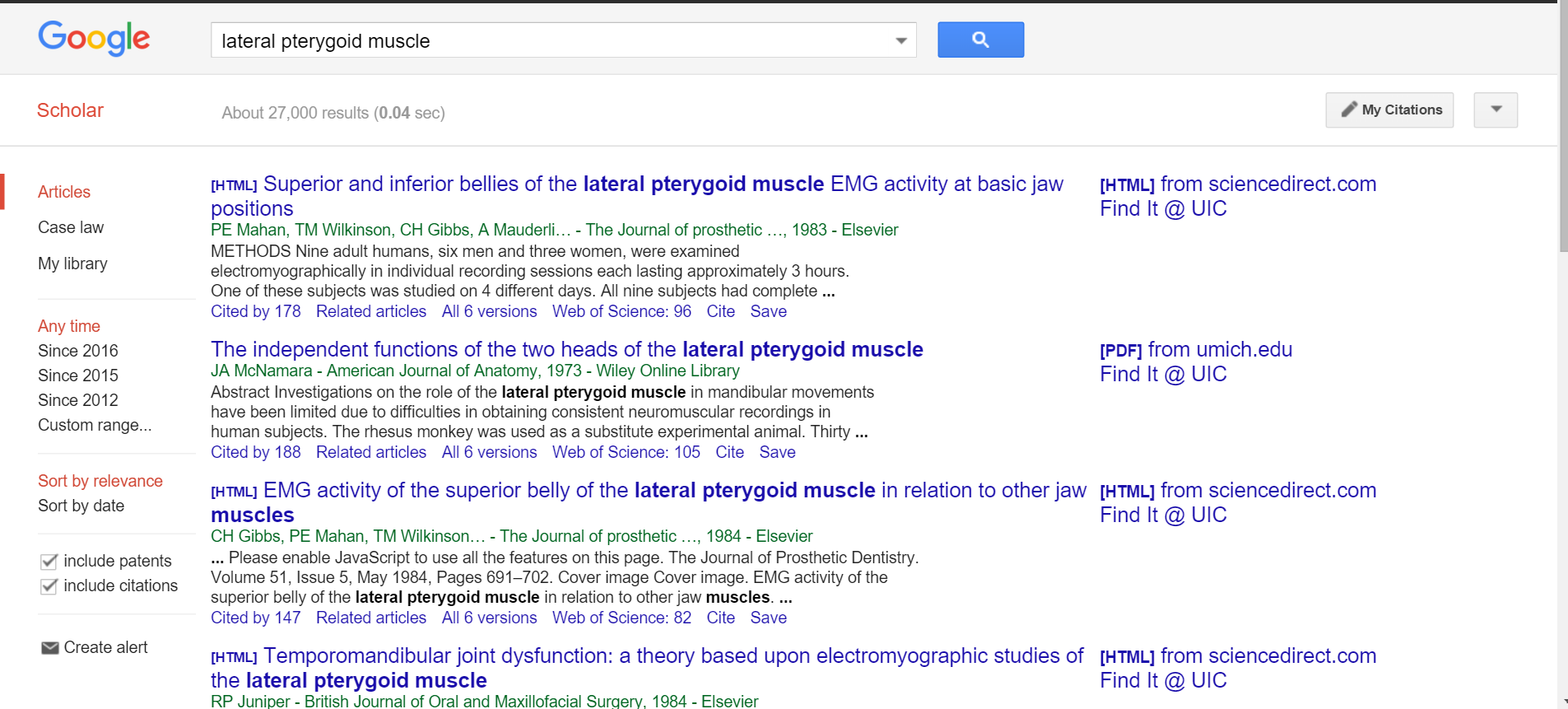

Supplement: S3 Fig — Only the first page is displayed. (PNG) [file pone.0149102.s003.png]

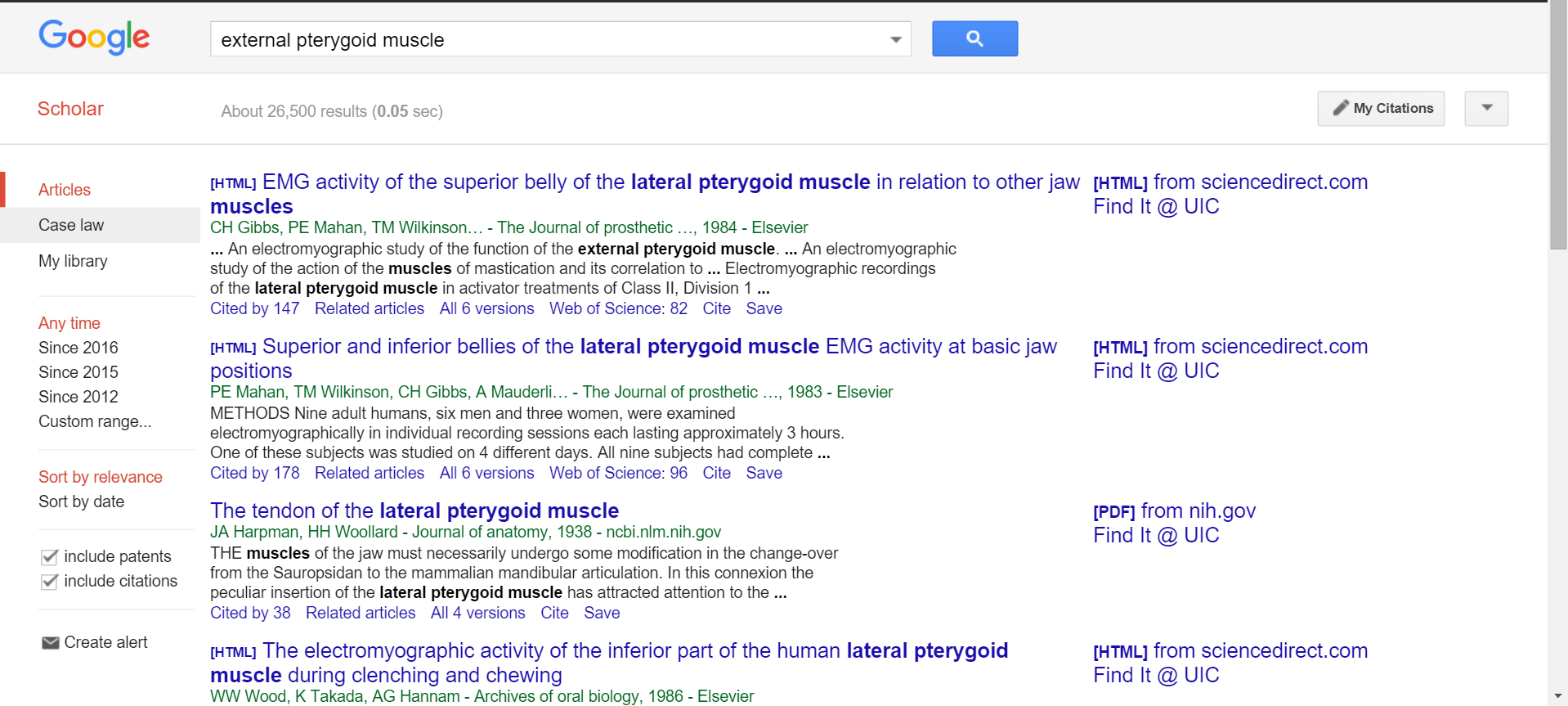

Supplement: S4 Fig — Only the first page is displayed. (PNG) [file pone.0149102.s004.png]

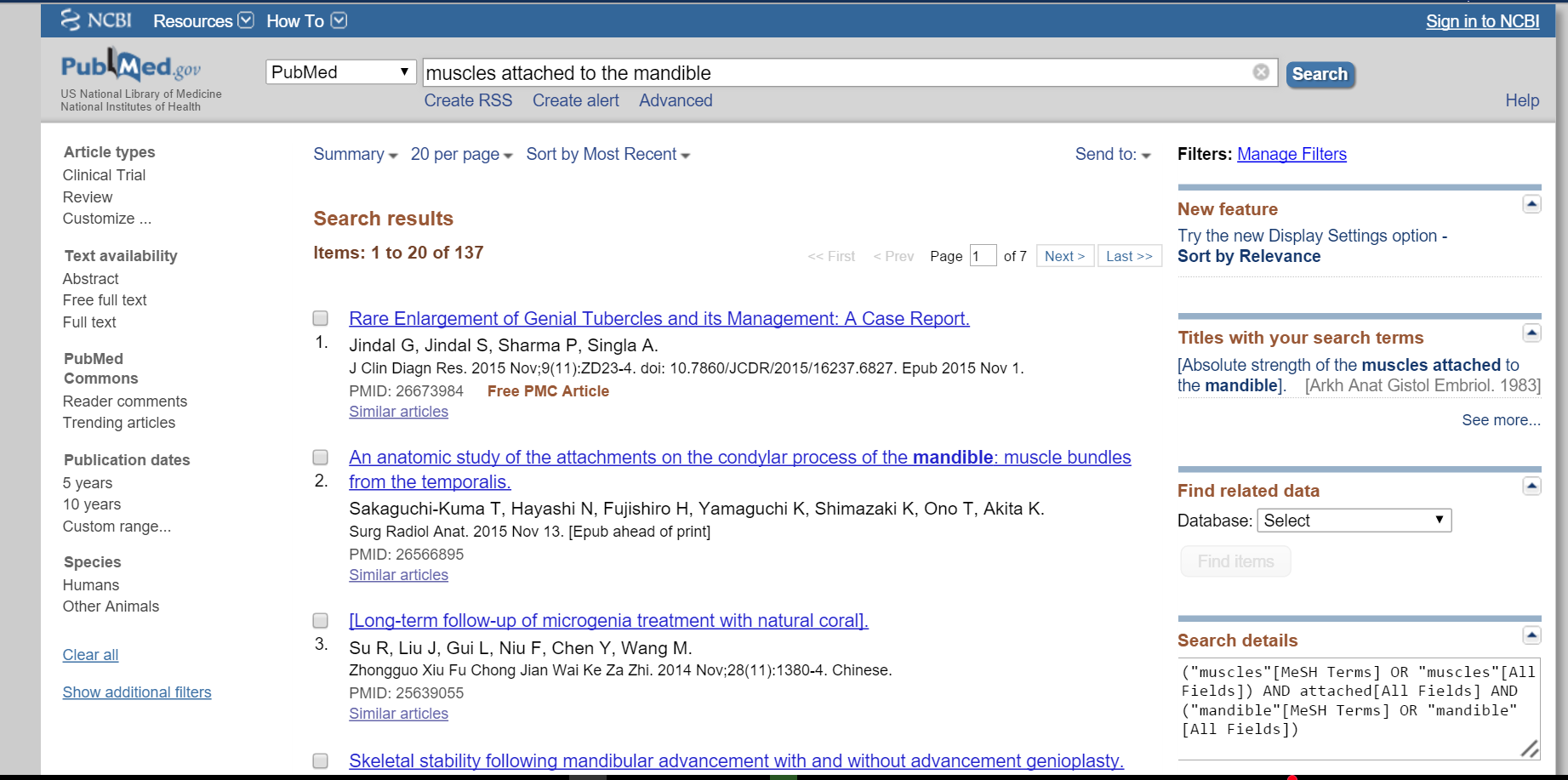

Supplement: S5 Fig — Only the first page of results are displayed. (PNG) [file pone.0149102.s005.png]

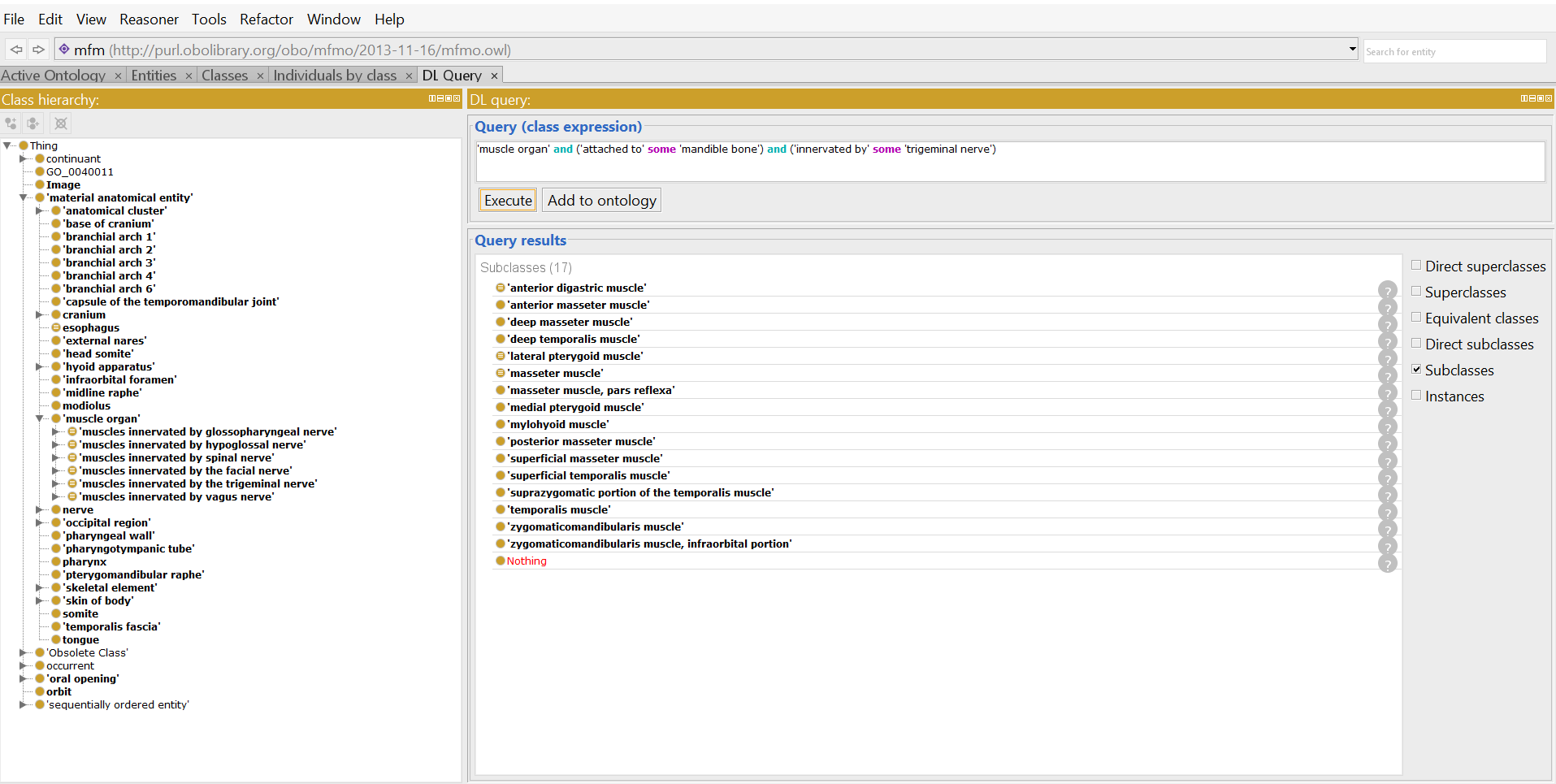

Supplement: S6 Fig — (PNG) [file pone.0149102.s006.png]

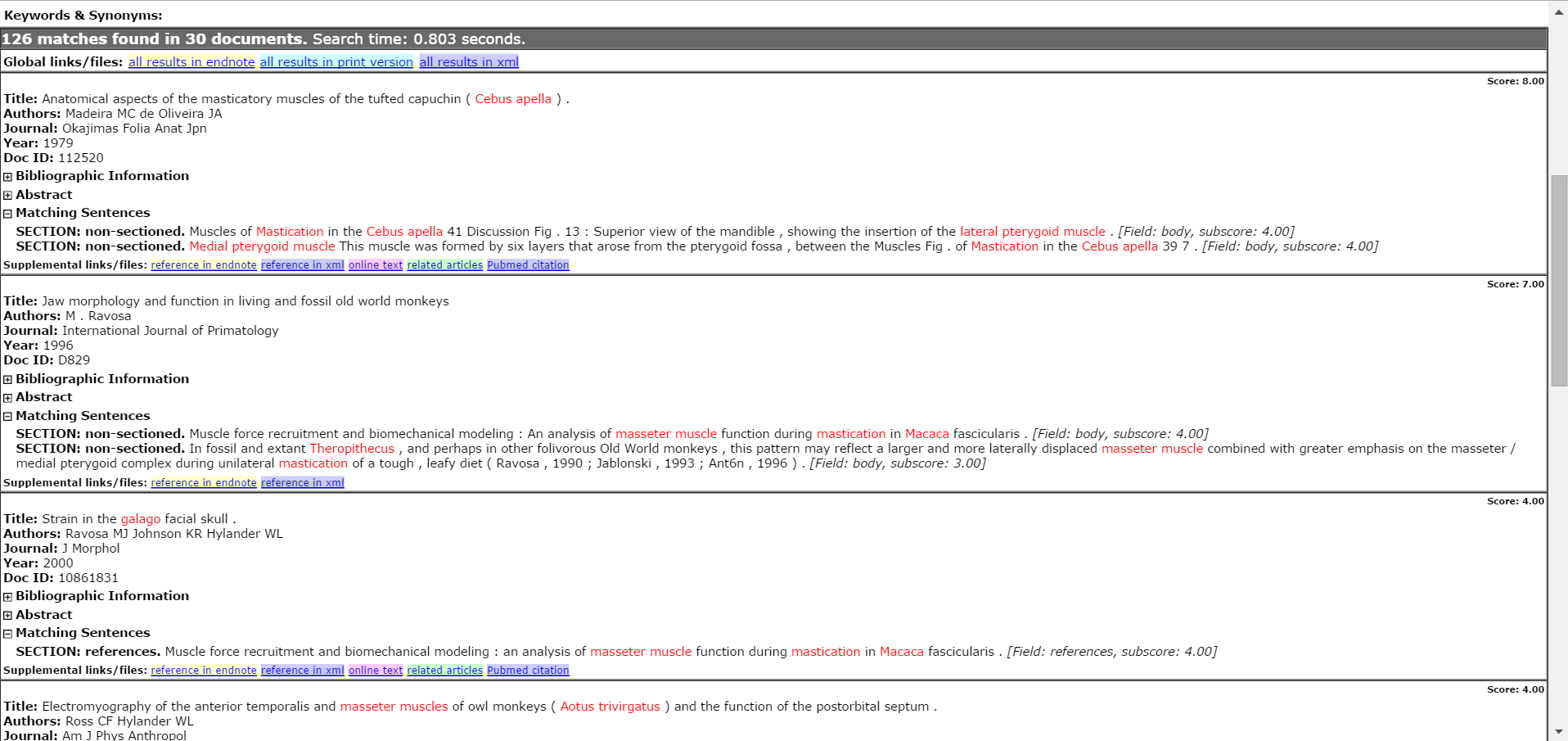

Supplement: S7 Fig — Only the first page of results are displayed. (PNG) [file pone.0149102.s007.png]
